# P-1146. Wastewater-Based Epidemiology for Emerging Pathogens in a Northern California Community Hospital, November 2024 to April 2025

**DOI:** 10.1093/ofid/ofaf695.1340

**Published:** 2026-01-11

**Authors:** Erika P Viana-Cardenas, Guillermo Rodriguez-Nava, Alessandro Zulli, Mingjun Jiang, Sehee Jong, Wajeeha Tariq, Eugenia Miranti, Mindy M Sampson, Nida Subhani, Alexandria Boehm, Jorge Salinas

**Affiliations:** Stanford University, Palo Alto, California; Stanford University School of Medicine, Stanford, California; Stanford University, Palo Alto, California; Stanford University, Palo Alto, California; Stanford University, Palo Alto, California; Stanford University, Palo Alto, California; Stanford Medicine, Stanford, CA; Stanford University, Palo Alto, California; Stanford Health care TriValley, Pleasanton, California; Stanford University, Palo Alto, California; Stanford University, Palo Alto, California

## Abstract

**Background:**

Emerging infections can lead to hospital outbreaks, and have the potential to cross borders and impact global health. While wastewater-based epidemiology (WBE) has been effectively utilized to monitor respiratory viral circulation at the community level, its application in hospital settings remains largely unexplored. We used WBE to gain insights into emerging pathogen circulation within a hospital environment.

**Figure:**
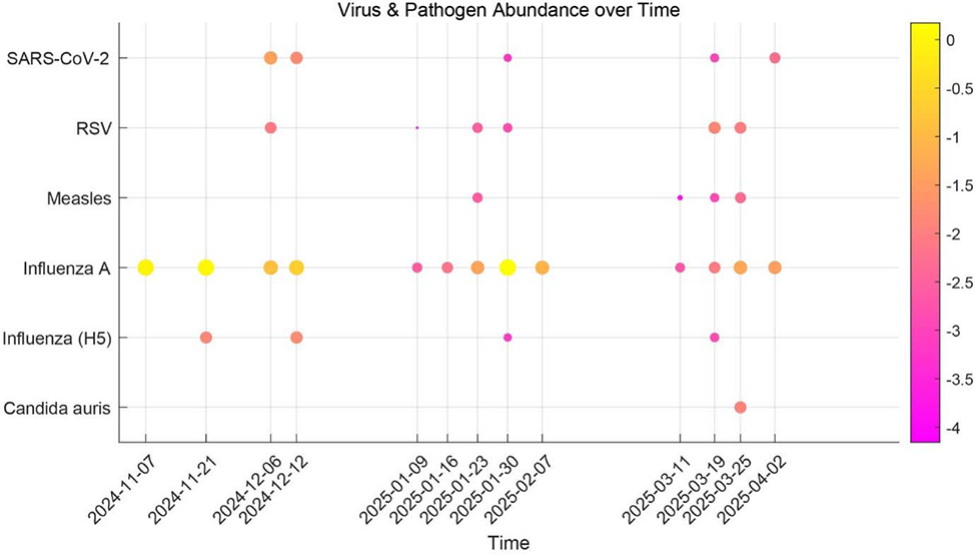
Emerging Pathogens detected in Wastewater from Tri-valley Hospital

**Methods:**

We collected 500 ml weekly composite wastewater samples using a Teledyne ISCO 5800™ autosampler from Tri-Valley Hospital, which is a 200-bed community hospital in Northern California. Samples were processed within 48 hours, with solids separated through centrifugation. Nucleic acids were extracted using column-based kit Zymo R2002. Droplet digital PCR was performed to detect viral nucleic acid using assays for viral genes detection developed and tested by the WastewaterSCAN program. We tested the hospital wastewater samples for presence of SARS-CoV-2, Respiratory syncytial virus (RSV), Measles, Influenza A, Influenza A subtype H5, and *Candida auris*. Data was analyzed using the QX Manager Software Version 2.2 (BioRad, #168032).

**Results:**

Influenza A virus was consistently detected during the observation period, while SARS-CoV-2 and RSV had more variability and less concentration (Figure). Interestingly, the presence of measles virus and Influenza A subtype H5 genetic material was detected in at least three samples. *Candida auris* was absent in most samples tested, except one week. No human cases of Measles, Influenza A subtype H5 or *Candida auris* were detected in Tri-valley Hospital during the observation period.

**Conclusion:**

WBE was reflective of classical epidemiology for SARS-CoV-2, RSV and Influenza A. The detection of Measles and Influenza A subtype H5 despite lack of clinical cases may signify this strategy has a high sensitivity and may yield viral shedding post vaccination (measles) or consumption of dairy products (Influenza A subtype H5). The detection of *Candida auris* in wastewater may represent an undetected case in the hospital.

**Disclosures:**

All Authors: No reported disclosures

